# Supplementary figures and images for: Evaluation of Antimicrobial and Anti-Biofilm Formation Activities of Novel Poly(vinyl alcohol) Hydrogels Reinforced with Crosslinked Chitosan and Silver Nano-Particles
Source: Polymers (Basel). 2022 Apr 16;14(8):1619. doi: 10.3390/polym14081619 (PMC9026349; doi:10.3390/polym14081619)

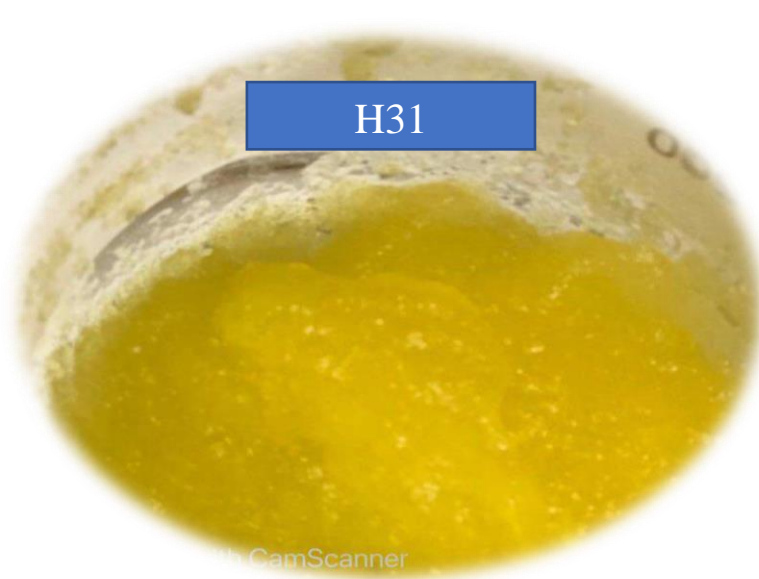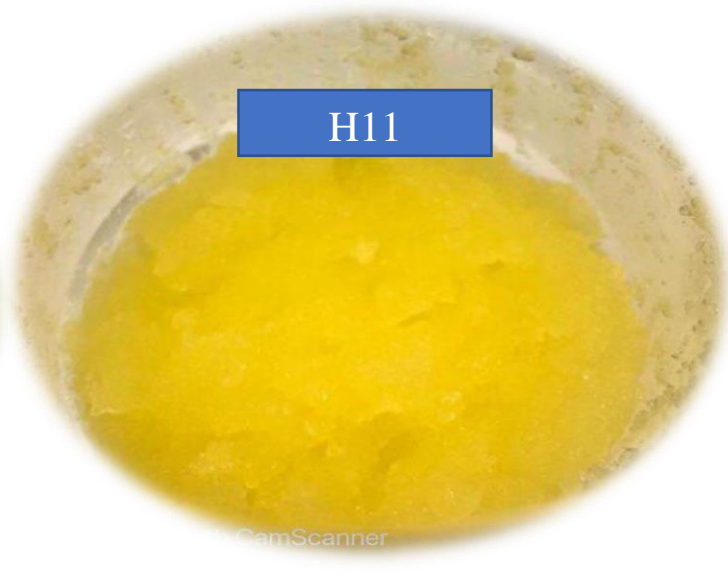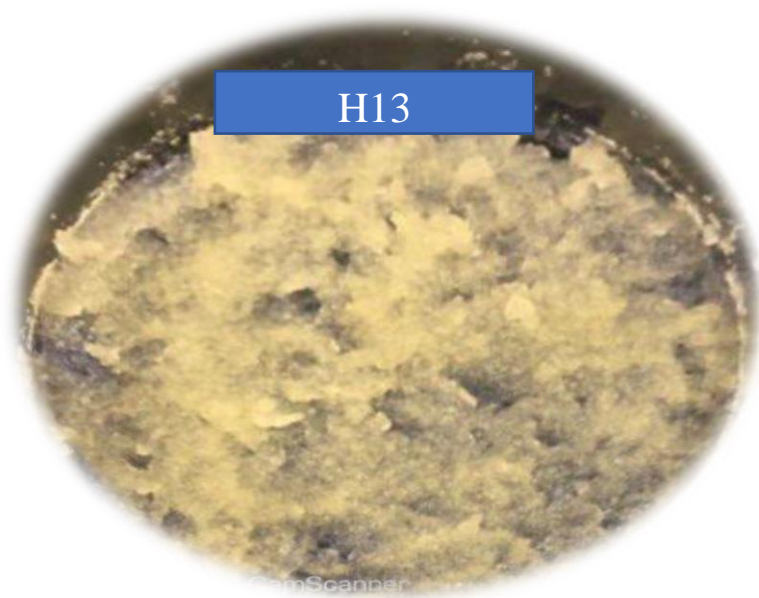

#### Online supplemental File

Normal optical images of the novel hydrogels.

Supplement: Supplementary file 1 [file polymers-14-01619-s001.zip › polymers-1666546-supplementary.pdf]
